# On the Use of Water in the Treatment of Diseases of the Skin

**Published:** 1880-01

**Authors:** L. Duncan Bulkley

**Affiliations:** Physician to the Skin Department, Demilt Dispensary, New York; Attending Physician for Skin and Venereal Diseases at the New York Hospital, Out-Patient Department, etc.


### ARTICLE III.

ON THE USE OF WATER IN THE TREATMENT OF DISEASES OF THE SKIN, by L. DUNCAN BULKLEY, A.M., M.D., Physician to the Skin Department, Demilt Dispensary, New York; Attending Physician for Skin and Venereal Diseases at the New York Hospital, Out-Patient Department, etc. Read before the American Medical Association, at Atlanta, Ga., May 7, 1879.

Water is by no means an indifferent agent as regards its influence upon the skin, either in health or disease; its right use is capable of much good, its abuse is equally capable of much evil. As it is an agent which is ever at hand, and one which instinct suggests the employment of, it is pretty certain to be used in most diseases of the skin by the laity as well as by the physician, and it is well therefore for the latter to have definite views in regard to its value when properly employed and its harm when wrongly applied.

The employment of water to the cutaneous surface may be considered in reference to its use in health as preventive of disease, and its use when the skin is the subject of disease; in the latter case it may be studied both in regard to its general use to the healthy parts and its employment upon parts affected.

In regard to the application of water in health there is little question in my mind that abundant bathing serves as a great preventive, both of cutaneous diseases and of systemic disorders, although Hebra not long ago gave quite the contrary impression in his article upon the use of baths. The skin whose dried excretions are frequently removed and whose circulation is quickened by reasonable bathing, undoubtedly is in a more healthy state and is better capable of performing its functions than one whose sluggish action is favored by neglect, and whose epidermal covering is allowed to adhere until rubbed off in a dry state by the clothing, even though Hebra does argue that skin diseases are no more common among those who may be truly said never to bathe the whole body, as is seen among some eastern peoples, than among bathers.

In health then, I regard the daily use of the cold or tepid sponge bath, followed by active friction, as one of the greatest safeguards against disease, both of the integument itself and of the system at large. In regard to warm baths, they have their function, and as a nervous sedative they may be of real service to certain persons, and when the daily tepid or cold ablution of the entire surface is not performed, they should be employed at stated intervals for the removal of effete matter from the skin; once a week, as is so commonly practiced, usually suffices. After a warm bath, a cold douche, as with a shower, or with a sponge in a pail of cold water, greatly enhances the power of the bath as a quickener of cutaneous circulation, and diminishes the danger of catching cold.

The subject of the use of the Turkish and Russian baths and their modifications in health, is too large an one to enter upon at the present time, and in a measure foreign to the subject of the paper. Suffice to say, they are continually abused in health, not only as relates to any sudden accidents which may and occasionally do result to those in peculiar conditions, but also as to their repeated use, which, like any powerful stimulant, may be and constantly is followed by a reaction whose ultimate effect may be very bad. They are not the panacea for all bodily ills, they are not the balm of life, securing immunity from disease, indeed, no small share of cases of diseases of the skin in private practice occur in those who have previously employed these steam or hot air baths to a greater or less degree. Their proper use in certain diseased conditions of the skin will be referred to later.

The use of water to the skin in its diseased state, may be considered under several heads, as follows:

1. Common water baths, including river and sea bathing.
2. Ablutions, bathings or soakings, with hot and cold water.
3. Cold and hot packing: water cure.
4. Vapor and hot air baths.
5. Medicated water baths.
6. Natural mineral springs.

1. The ordinary warm bath, as before mentioned, has for its function the removing of effete matter and softening the skin, and equalizing the circulation. It may thus have a favorable action in disease and may be used with advantage in chronic scaly eczema, psoriasis, ichthyosis, also in lupus and in obstinate ulcerative syphilis. This warm bath has been long used by Hebra continuously in certain cases, that is the patient remaining in the water for weeks or even months, the temperature being maintained by steam or a current of hot water, the water being also changed more or less continuously. In these the patient eats and sleeps in the bath upon a proper mattrass, only leaving the bath to empty the bowels. These have been found to be of principal service in burns, pemphigus foliaceus, phagedæna, etc., as I have myself witnessed, and are of really great value. In some instances patients have remained in them for many months consecutively.

But ordinary warm water baths cannot always be advised with impunity in all diseases of the skin; acute eczema, or even more sub-acute, exuding forms of this disease will be found to be aggravated by the contact of simple water. Urticaria will also be rather irritated, as will indeed most of the inflammatory affections of the skin. It is well to remember when baths are given to a patient with a pruritic skin, to give a caution against too great friction afterwards, as this not infrequently more than balances any soothing effect of the bath, and the irritation afterwards may be very great.

In regard to river and lake bathing, the reaction and the exposure to the air will aggravate most skin diseases. Sea bathing is of service in psoriasis, and warm baths of sea water have been followed, in my experience, with very beneficial results in this disease; it is a remedy which I resort to a good deal during the summer months. But sea bathing is decidedly harmful in most cases of eczema, as I have repeatedly witnessed, although I have heard of a few instances where the disease was very chronic and the eruption very indolent, in which sea bathing was said to have cured the cases. But I am very cautious about allowing eczema patients either to bathe in the sea or to spend much time at the sea shore. The same is true of acne, and every autumn I see many, many cases where the sea influence has greatly aggravated the eruption, and many where the eruption has appeared for the first time while by the sea side; sea voyages generally act unfavorably in acne. Sea bathing sometimes is of much service in chronic urticaria, but in most of the acute inflammatory affections of the skin its use is to be prohibited.

2. In regard to ablutions or washings and bathings or soakings with hot and cold water, much may be said. The general tendency is to bathe or wash a diseased part for the sake of cleanliness, and this will pretty certainly be done, whether the physician directs it or not, and often in a manner greatly injurious to the lesion. It is a most common practice to wash eczema, and especially do we often see the eczema of children washed diligently, often several times daily, the mothers saying that they find it "impossible to keep it clean." Now, in my opinion, an eczematous surface should be washed very seldom and that only by special direction from the physician. Nature seeks to make a protective covering with the exudate; this is continually removed by washing, the process is repeated and cure is most certainly retarded. If more time be now given for the formation of a more resisting epidermal covering, occasional washings, serving to remove superfluous exudation and thus to allow the astringent used to come into contact with the diseased surface, repair will take place more speedily. I have repeatedly witnessed eczema in children get well under the same treatment previously employed by the physician calling the consultation, when only the frequent washings were omitted.

The same is true of eczema in the adult; as an instance of the effect of water in causing and prolonging an eczema, we may mention the case of the eruption when it occurs on the hands of wash-women, or those whose occupation compels frequent wetting of the hands. It is next to impossible to cure some of these cases, as in bar-tenders, waiters in hotels, as well as wash-women without a cessation of the occupation, and just so impossible is it to cure certain cases of eczema while frequent washing is practiced voluntarily.

But washing is indicated at times, and some judgment is necessary to determine just when this should be practiced. In eczema of the scalp it is rarely best to wash the head more than once or twice a week, and here, even more than elsewhere, it is specially necessary to reapply the ointment or other dressing *immediately* after the cleansing, before the denuded surface has had time to become again covered with the impenetrable coating which quickly forms.

Proper use of water is also of much service in certain patches of chronic eczema, when there is much thickening of the skin, with oft-times desperate itching. Such patches may be washed daily with advantage, and sometimes they will bear very great stimulation. Thus, taking the sapo-viridis and a bit of flannel and water, very sharp friction may be made for several minutes, after which the part is to be washed off clean with warm water and immediately covered with the appropriate dressing; the stimulation may even be carried so far as to use a brush, and I well remember a plasterer at Demilt Dispensary, who scrubbed the backs of the hands with a common floor scrubbing-brush and soft-soap and water until they bled. The result of this active treatment was to remove a greatly thickened eczema of many years standing, so effectually that, although he has continued his occupation and I have seen him repeatedly during the past four years with eczema on the sides of the hands and fingers, the backs of his hands, which were the seat of his severe attack and energetic treatment, have remained perfectly smooth and healthy.

Hot water is sometimes of the very greatest value in eczema. In eczema of the anus often nothing gives so much relief as holding a cloth dipped in water, as hot as can be borne, against the parts and repeating the application two or three times; the part being then covered with the dressing appropriate to the case; the same is of service in eczema of the vulva. In eczema of the scrotum I frequently direct that the part shall be suspended for a few moments in a cup of very hot water before other application is made. Simple pruritus of these parts is also often greatly relieved by these hot applications.

Chronic eczema of the palms of the hands, where the surface is hard, dry, fissured, often shiny, and the hands well nigh useless, will sometimes seem almost to melt away under the daily soaking of the palms on the surface of a basin of scalding hot water, followed by diachylon or other ointment. Eczema of the ends of the fingers and of the nails sometimes yields to this after all other measures have failed.

Onychia, both where there is and where there is not an apparent eczematous element, is also very greatly benefited by these soakings in very hot water, accompanied and followed by other appropriate measures.

Indolent ulcers of the legs take on active changes and often cicatrize rapidly under the powerful stimulation of the alternate application of a cloth dipped in very hot water, followed instantly by one taken out of a vessel of very cold water.

Very striking results are often obtained from the use of hot water in some of the forms of acne. It is applied by means of a cloth, as a handkerchief, dipped in the hot water and held to the face until the heat of the cloth has passed off, when the performance is repeated two or three times for a period of not over three minutes to five altogether; a long soaking of the face in water which is not hot enough will aggravate the eruption, but the reaction following the brief application of very hot water, is often very remarkable. After multiple scarification of the pustules and papules of acne, prolonged bathing with tepid water is of service in encouraging the bleeding, which otherwise always tends to cease sooner than is desired.

In certain cases of chronic erythema, where the congestion resists other measures, the alternate application of cloths dipped in very hot and very cold water, serves to break up the capillary stasis. On the other hand, repeated washing of ulcerative surfaces will often be quite sufficient to prevent their healing; this is often seen in ulcerative syphilides, which will sometimes resist the proper internal medication as long as repeated washings are persisted in, and yields to it almost immediately when cotton batting is applied and left undisturbed. Varicose ulcers of the leg are not infrequently kept from healing by the too diligent cleansing which patients are ever ready to bestow.

3. Cold and hot packing in wet sheets, as practiced in the water cures, is of a certain value as a remedy in diseases of the skin. The results which sometimes occur, as boils on the surface, are to be deprecated, and are not, as is popularly supposed, either a good sign, or a good element in the treatment; we of course no longer believe that there is a "materies peccans" which needs to be eliminated. But the wet pack has served well in the hands of Hebra in the treatment of acute psoriasis, also in acute general eczema. The packing is made twice daily, for for several hours, morning and evening.

This is most conveniently accomplished by placing two blankets lengthwise upon a bed, and over them a sheet dipped in cold water. The patient then lies naked upon this, which is closely folded over him up to the chin, and the blankets are then wrapped closely around, and the whole done up with bands, so that the patient is immovably fixed, helpless indeed. The first sensation is that of a pleasant glow, and before long perspiration ensues, which should be encouraged by draughts of water frequently given; the packing lasts from two to five hours. It should never be forgotten to place an urinal between the thighs of the patient before envelopment.

Under this treatment the scales of psoriasis disappear, and the red patches daily become less visible. Few patients in this country will submit to this treatment, but when it is desired to remove the existing eruption in the shortest possible time, it is of value. Packing is not of service in many affections of the skin, and should seldom be prescribed; although in the water-cure establishments all eruptions are submitted to this course. The profession need more accurate scientific information in regard to the precise effects and the therapeutic indications of this powerful remedy.

4. Vapor and hot air baths, and their modifications, may play a not inconsiderable part in the treatment of certain cutaneous diseases, although from their expense and the difficulty of their application at home, and from the fact that most of the establishments where they are administered are in the hands of ignorant, unprofessional persons, they are not employed to the extent which their real value would indicate.

While there are undoubtedly differences in the mode of action and in the results obtained from the Russian or vapor and the Turkish or hot air baths, I am not aware that any distinctive therapeutical indications between them, or effects from one more than the other have been scientifically noted in diseases of the skin. The Turkish or hot air bath, with the subsequent cold douche, is that generally preferred and undoubtedly is the safer. It is of a certain value in psoriasis, and of some service in chronic eczema, also in chronic urticaria, lichen and ichthyosis, but the results obtained are generally rather disappointing, and I must acknowledge that I use them comparatively seldom.

Certain modifications of the steam or vapor bath are, however, oftimes of considerable aid in treating diseases of the skin. Under this head come the sulphur vapor and mercurial vapor baths, which, as is well known, have great repute in this line of practice. They consist in a steam bath to all the body, save the head, combined with sulphur or mercury volatilized by heat. The value of the mercurial bath, in many cases of syphilis, need not be dwelt upon ; its use should be strictly confined to cases where this diagnosis is certain. The indiscriminate use of a mercurial or sulphur vapor bath when the skin is affected is highly reprehensible.

The real utility of sulphur vapor baths in diseases of the skin is in a measure still *sub judice*. Their anti-parasitic value is fairly positive. If well used they will cure scabies, phtheiriasis and the vegetable parasitic diseases. But even in these the irritation occasioned by them is sometimes so great that the artificial eruption produced quite masks that of the disease proper and prevents their continuance. Sulphur vapor baths are also of some value in psoriasis, and occasionally the eruption will seem to yield to them, but quite as often they are powerless, as I have frequently witnessed. Chronic, more or less generalized papular eczema, may be much benefited by sulphur vapor baths, given every other day or so; but here care must be exercised, for this very condition may be entirely caused by their use.

Mr. J. L. Milton, of London, has arranged a convenient apparatus by means of which vapor baths, with or without the addition of sulphur or mercury vapor, may be administered at the patient's house with comparatively little expense. It consists of a series of hoops over which a thin rubber or gutta percha covering is stretched, which, when the patient is seated on a chair is gathered around the neck and reaches to the ground ; a second cloak of coarse flannel is placed within the rubber and over the hoops. The patient sitting naked on the chair, with the Mackintosh closely drawn around the neck, a lamp with a steam generator is placed on the floor beneath the chair ; after the body has been submitted to the steam for some minutes, if it is so desired, the sulphur or mercury is introduced on a receptacle over the lamp, placed in the centre of the pan of boiling water, and continued for a few minutes longer. By means of this apparatus steam baths may be given with advantage in a number of cutaneous affections; acne, chronic eczema, lichen, psoriasis, etc., and when it is desired, a cold dash afterward makes of it a very decent substitute for the ordinary Turkish or Russian bath, according as steam is used or simply hot air; this can, moreover, be employed in localities where these are not accessible, or in cases where it is not desirable to have the patient leave the house. I have also had used a wooden box, constructed at home, somewhat like those employed at the establishments, in which the patient sits with the heat, which may be given by an ordinary low gas stove, beneath the chair. This can be made by any carpenter and is very inexpensive.

Mention must be made here of the vapor bath or douche applied locally in acne, as recommended so largely by French writers. It consists in holding the face over a basin of standing water, with the head and basin enveloped in a blanket or other covering. This plan answers very well in many cases, and greatly relieves the congestion, but the indications for it are better fulfilled, I think, in the method of soaking the face with hot water as previously described.

5. Medicated water baths. This is a very important division of our subject, and should always be considered in dealing with diseases of the skin. Not only is this an important matter in reference to the direct action of the bath upon the *diseased* skin, but the general medicated bath is to be considered in regard to its effect upon the general health, which may be very considerably modified thereby. Experimental investigations have shown very conclusively that under the frequent use of water to the whole surface, assimilation and disintegration proceed more rapidly than without it. In certain skin diseases, therefore, where these processes are slowly or imperfectly performed, the warm bath, especially when holding alkalies in solution, does much to improve the general health and to remove diseases of the skin, even from locations which are not submitted to the bath. Thus I have found many cases of acne to make much greater and more rapid improvement when alkaline baths were added to the measures previously employed; the same is true of some cases of eczema of the face or scalp.

Many ways of medicating the water to be employed in the bath have been suggested which need not be mentioned here, inasmuch as practically but few are employed in daily practice, and the indications for the use of the various kinds of baths have never been laid down with any clearness or precision; and I regret that time forbids my entering more deeply into the subject at this time.

My most common medication for baths consists of carbonate of potassa, carbonate of soda, and powdered borax, four, two and one, ounces of each, respectively, together with from one-quarter to one-half pound of starch to a bath of thirty gallons. This is a mild alkaline bath, is decidedly soothing to most skins, and acts very favorably on sub-acute eczema and urticaria; it may be used much stronger, as in psoriasis, ichthyosis, prurigo, etc. The bath is to be given at bed time, at a temperature of from 87° to 95° Fahrenheit, the patient remaining in the water fifteen or twenty minutes. On coming out, if the skin is pruritic it is not to be rubbed, but dried with a heated sheet, and all diseased surfaces are to be covered at once with a suitable ointment. I find it very grateful to the patient to have the entire surface lightly annointed with the glycerite of starch, or cosmoline, to which a small quantity of carbolic acid, five to ten grains to the ounce, may be added, if there is much general itching.

Such medication of the bath may be modified to suit certain circumstances; thus, the starch may sometimes be replaced by gelatin, a half a pound to the bath, well boiled; or glycerine, four to ten ounces to the bath, will make it more soothing to some skins. Sometimes the soda and potash prove too drying,and the quantity of borax may be increased and these diminished.

It is difficult to give in a few words the exact indications for the use of these alkaline and starch baths, but in general I may repeat that they are soothing to a pruritic skin and serve admirably to promote the processes of life. Sub-acute and chronic eczema are almost always greatly benefited by such a bath, taken at bed time, twice or three times a week, or even every night, if not too debilitating.

Sulphuret of potassium has been frequently used in baths, especially to relieve itching, but I do not employ it except as used in the artificial Barèges baths, which may be obtained at some of the bathing establishments; baths made with sulphuret of potassium should be given in a wooden tub, as the salt rapidly attacks metal lined tubs, if in a strong solution. I have seen a number of instances of cutaneous eruptions where sulphuret of potassium baths had been used to the great aggravation of the disease, and cannot give any indications for the employment of this kind of bath, although the books mention it as valuable.

Occasionally benefit will be derived from the use of other substances in solution in baths. Thus, Hebra makes very considerable use of a bi-chloride of mercury bath, in syphilis, lupus and prurigo; nitric acid, or nitro-muriatic acid baths have been used with advantage in jaundice, and also in other diseases, especially to relieve itching, and to promote cutaneous action. Sometimes vegetable substances may be added to a bath, in certain diseases of the skin, as conium, opium, etc., for a sedative effect and the relief of pain; or astringents, as oak bark or tannin, for their particular effects.

The common warm, or an alkaline bath is sometimes given with much effect after the application of tar; thus, in chronic and much thickened eczema, the part is tarred over, and the patient enters a bath, remaining there 15 or 20 minutes; this is a favorite plan with Hebra.

6. Finally, the natural mineral springs should never be forgotten in the treatment of the obstinate cutaneous affections, although their efficacy is, in my opinion, greatly over-rated. The subject is too large an one to enter upon on the present occasion and will be deferred to another opportunity. Suffice it to say here, that while mineral springs are of much real service in many diseases of the skin, their use is not to he rashly advised without a definite knowledge of what is to be accomplished; they are to be prescribed, as any other remedy, to fulfill definite indications; certain springs, while beneficial to certain eruptions, are just as surely harmless or inefficatious in others. The habit, therefore, of sending a patient to particular mineral springs simply because affected with a disease of the cutaneous surface, is as irrational as it would be to send one indifferently to Florida, Colorado, Minnesota or the Adirondacks, because some organ within the chest happened to be diseased, without any idea of the nature of the malady or the object to be effected by the particular climate in question; or one could quite as reasonably send one to a drug-shop because sick, without giving a prescription for the remedy required.

There is certainly great need of more definite and reliable scientific information, based on recorded facts, in reference to the vast resources of our wide country in the matter of natural mineral waters, and I cannot forbear the hope that a hydrological committee may be formed in this Association for such investigation. A not inconsiderable amount of information on the subject has come to my knowledge within the past ten years and I shall hope on another occasion to collect it from my notes of cases and to present it before you. I can premise in regard to it, however, that much of it is of a negative character, for I see multitudes of patients who have received but slight or temporary improvement or have failed entirely of benefit from a wrong use of mineral waters in diseases of the skin.

